# Development of a hydrophilic transdermal patch for combined delivery of sumatriptan and metoclopramide in migraine therapy

**DOI:** 10.1007/s13346-025-01874-0

**Published:** 2025-05-20

**Authors:** Ariana Radmard, Ajay K. Banga

**Affiliations:** Center for Drug Delivery Research, Department of Pharmaceutical Sciences, College of Pharmacy, Mercer University, Atlanta, GA 30341 USA

**Keywords:** Hydrophilic transdermal patch, Migraine treatment, Sumatriptan-metoclopramide combination, Drug permeability enhancement, In vitro permeation

## Abstract

Migraine, a prevalent neurological disorder, is known to significantly impact patients' quality of life. The effectiveness of oral medications is often hindered by nausea and vomiting, common migraine symptoms. In this study, a transdermal patch for the co-delivery of sumatriptan succinate and metoclopramide HCl was developed and evaluated, to offer a patient-friendly alternative for migraine management. This study evaluated the impact of chemical enhancers and hydrophilic formulations on drug permeation using dermatomed porcine ear skin. A combination of 25% w/w propylene glycol (PG) and 10% w/w dimethyl isosorbide (DMI) significantly enhanced the permeation of both drugs. Among four hydrophilic patch formulations, a matrix composed of a 1:3 ratio of polyvinylpyrrolidone (PVP) to hydroxypropyl methylcellulose (HPMC) demonstrated optimal drug delivery. Characterization tests, including coat weight, drug content uniformity, tack testing, and slide crystallization, were performed, all patches exhibited acceptable physical properties, including uniform drug content. The target therapeutic dose, equivalent to 4 mg subcutaneous injection of sumatriptan succinate and 10 mg oral metoclopramide HCL, was achieved within 8 h using a 60 cm^2^ patch of the optimized formulation. Based on these findings, the developed transdermal patch could serve as a promising alternative for migraine management, potentially improving patient compliance and therapeutic outcomes.

**Graphical abstract:**

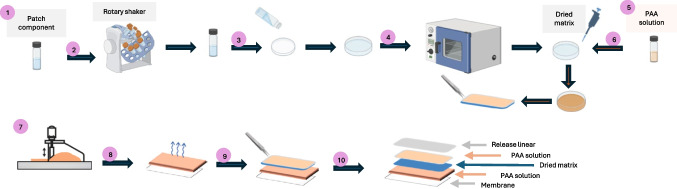

## Introduction

Migraine, a prevalent neurological disorder affecting over a billion people worldwide, is characterized by recurrent, moderate to severe headaches [1]. These headaches are often throbbing and unilateral but can also be bilateral or accompanied by pain in the face or neck (Migraine | NHS Inform, 2.). The pain, which often worsens with movement, can significantly impair daily functioning, disrupting work, school, and social activities [3, 4]. As a result, this not only places a heavy burden on individuals and their families but also strains healthcare systems and reduces economic productivity [5]. The global prevalence of migraine is estimated at 15–18%, making it a leading cause of disability, particularly among women [3]. While the exact cause of migraine is unknown, it's believed to stem from abnormal brain activity affecting nerve signals, chemicals, and blood vessels [2]. Several factors can increase the risk of migraines. These include age, a history of head trauma, and lower socioeconomic status. Substance overuse and various lifestyle factors—such as chronic stress, sleep disorders, and obesity—also play a role. In addition, certain pain syndromes and medical conditions, particularly those associated with pro-inflammatory or pro-thrombotic states, can further elevate the risk [3]. Several treatment options exist for migraine management, including oral, nasal, and injectable formulations; however, each comes with its own set of limitations in terms of efficacy, patient compliance, and tolerability.

One of the most common methods for treating migraines involves the administration of oral medications. However, a significant problem with current oral migraine treatments is that they often fail due to nausea and vomiting, which are common symptoms experienced by a majority of migraine patients. In fact, studies indicate that nausea affects approximately 60–95% of patients, with 30% experiencing it during every attack, while vogiting is reported by 50–70% of patients, with nearly one-third experiencing it in the majority of attacks. Consequently, these gastrointestinal symptoms can significantly interfere with oral medication intake, leading to reduced drug absorption and delayed relief. For instance, approximately 30.5% of patients with nausea and 42.2% of those with vomiting reported that these symptoms hindered their ability to take oral migraine medications [5, 6]. To address these challenges, non-oral formulations like nasal sprays and injectable treatments have been developed. Nevertheless, while nasal sprays offer a bypass from the gastrointestinal tract, their effectiveness is limited by low bioavailability (~ 17%), and 25% of users report an unpleasant taste depending on the dose. Similarly, subcutaneous injections, although offering higher bioavailability (~ 97%) and rapid onset of action, are less popular due to discomfort and potential adverse effects, such as injection site reactions (59%) and atypical sensations (42%) [7, 8]. Given these limitations, there's a need for alternative migraine therapies that are both effective and patient friendly.

Triptans, a class of drugs targeting serotonin receptors, have revolutionized migraine therapy. Sumatriptan, the first of this family, binds to and activates specific serotonin receptors, primarily the 5-HT1B and 5-HT1D subtypes, located on the smooth muscle cells of intracranial blood vessels and trigeminal nerve fibers. This activation induces vasoconstriction and is believed to play a key role in alleviating migraine pain [8, 9]. In many cases, patients report complete headache relief after using sumatriptan, while others experience substantial pain reduction that allows them to resume their daily activities(Sumatriptan (Transdermal Route)—Mayo Clinic, 2024.). Despite its benefits, sumatriptan does have limitations. Notably, it has low oral bioavailability, leading to inconsistent drug levels in the bloodstream and reduced effectiveness. Moreover, sumatriptan is rapidly eliminated from the body, which can lead to headache recurrence, especially in patients with frequent or severe migraines [9].

To further enhance migraine management, metoclopramide, a dopamine D2 receptor antagonist, has emerged as a valuable tool due to its dual properties of pain relief and nausea control [10]. Notably, one of metoclopramide's key benefits is its ability to alleviate gastric stasis, a common issue during migraine attacks, by promoting gastric dilation and relaxing the lower esophageal sphincter (LES). As a result, this action enhances the absorption of other medications, making it a valuable adjunctive therapy [11]. Several studies have, in fact, demonstrated the clinical efficacy of metoclopramide in treating acute migraines, with a meta-analysis pooling data from multiple studies finding that it significantly reduced headaches. Furthermore, it has been a standard therapy in some headache clinics, emphasizing its widespread use and reliability [12]. The intravenous route of administration for metoclopramide has yielded particularly positive results, with doses of 10 mg and 20 mg both showing significant improvement in headache outcomes. This makes metoclopramide an ideal option for migraine therapy, especially for patients who experience nausea and vomiting during attacks [13]. In addition, a clinical study highlighted the synergistic effect of combining sumatriptan with metoclopramide, demonstrating improved efficacy compared to using a triptan alone and providing significant relief for some migraine patients who previously failed to achieve adequate relief with sumatriptan [14].

Transdermal drug delivery systems (TDDS) have emerged as an alternative to oral and injectable drug delivery methods, ranking as the third-largest drug delivery system [15]. The skin’s accessibility, extensive surface area, and ability to bypass the gastrointestinal (GI) tract make it an appealing route for systemic drug delivery [16]. In particular, TDDS provides significant advantages by delivering drugs through the skin for local or systemic effects, addressing many limitations of traditional delivery methods. The non-invasive nature of TDDS, for example, eliminates the pain, risk of infection, and potential disease transmission associated with injections, making it a highly patient-friendly option. Additionally, TDDS allows for controlled and consistent drug release, minimizing fluctuations in drug levels and reducing the risk of side effects [17, 18]. Furthermore, unlike oral medications, TDDS bypasses hepatic first-pass metabolism and avoids degradation in the GI environment, improving bioavailability and preserving drug efficacy [16]. However, despite their advantages, transdermal drug delivery systems face significant limitations due to the skin's selective permeability. Only a small number of drugs, with properties like low molecular weight and moderate lipophilicity, can cross the skin barrier effectively. This makes the transdermal route unsuitable for many pharmaceutical agents, especially macromolecules which struggle to penetrate the stratum corneum at therapeutically relevant rates [19]. For drugs to enter the bloodstream through the transdermal route, they must pass through the skin's natural barriers, first penetrating the stratum corneum, the dense outermost layer of the skin, then moving into the epidermis and finally reaching the dermis. Transdermal patches are designed with specific layers, including a backing layer for protection, a drug reservoir or matrix in adhesive for controlled release and to keep the patch in contact with the skin, and a release liner [20]. These features make transdermal drug delivery systems an effective and innovative approach, improving therapeutic outcomes and increasing patient compliance.

In this study, we developed a hydrophilic transdermal patch to deliver a combination of sumatriptan succinate and metoclopramide hydrochloride (HCL). Their combination used for acute migraine treatment but, to the best of our knowledge, never previously formulated together in a single transdermal patch. Both sumatriptan succinate and metoclopramide HCL are hydrophilic drugs with log P values of 0.736 and 1.397, respectively, and exhibit high solubility in water. These physicochemical properties made them ideal candidates for incorporation into a hydrophilic patch formulation. We explored the effects of various chemical enhancers on drug delivery and investigated the impact of different hydrophilic patch formulations to optimize drug permeability. We successfully delivered a dose equivalent to 4 mg of subcutaneous injection of sumatriptan and 10 mg of oral metoclopramide simultaneously within 8 h through a 60 cm^2^ hydrophilic transdermal patch. This patch offers several clinical advantages over conventional oral and injectable dosage forms. Unlike oral tablets, its performance is not compromised by nausea and vomiting, common symptoms during migraine attacks. It also eliminates the need for needles, improving comfort and patient compliance. Furthermore, its ease of use and self-administration make it a more patient-friendly and accessible treatment option for managing migraines.

## Materials

Sumatriptan succinate purchased from Asta Tech (Bristol, PA, USA). Poly(ethylene glycol) (PEG 400), propylene glycol and HPLC-grade solvents were obtained from Fisher Scientific (Pittsburgh, PA, USA). Hydroxypropyl methylcellulose (HPMC) was gifted from The Dow Chemical Company (Midland, MI, USA). Metoclopramide HCL, carboxymethyl cellulose (CMC-Na), gelatin, d-mannitol, and 1-methyl-2-pyrrolidinone (NMP) were acquired from Sigma-Aldrich (St. Louis, MO, USA). Sodium polyacrylate was purchased from Ward's Science (Rochester, NY, USA). Poly(acrylic acid) (50 wt% and 25 wt%) was obtained from Thermo Scientific (MA, USA). Dimethyl isosorbide and isopropyl myristate were received as gift samples from Croda Inc. (Edison, NJ, USA). Glycerol was sourced from EMD Millipore Corp (Massachusetts, USA). Release liners (ScotchPak™ 9744, ScotchPak™ 1022, 9709 YDS sample roll SKU, and 27323 PET) and backing membranes (CoTran™ 9720, CoTran™ 9728, CoTran™ 9702, CoTran™ 9707, CoTran™ 9706, CoTran™ 9722, and ScotchPak™ 9733) were obtained as gift samples from 3M Company (St. Paul, MN, USA). Porcine ear skin was acquired from Animal Technologies (Tyler, TX, USA).

## Methods

### Quantitative analysis

A reverse-phase high-performance liquid chromatography (HPLC) method was developed to simultaneously detect sumatriptan succinate and metoclopramide HCL in the samples. The method was developed using an ODS-AQ SS 120 Å column (4.6 × 250 mm) with an injection volume of 10 µL and a flow rate of 1 mL/min. The mobile phase was composed of methanol with 0.3% THF as an organic phase and 10 mM potassium phosphate buffer at pH 4 as buffer phase, injected in a 40:60 ratio. The detection wavelength for both, was 279 nm. The method was validated through inter-day and intra-day accuracy and precision test, as well as linearity, limit of detection (LOD), and limit of quantification (LOQ).

### Saturation solubility

To determine the optimal donor concentration and the most suitable receptor solution for sumatriptan succinate and metoclopramide HCL, their saturation solubility was evaluated in various vehicles. First an excess amount of the drug added to the vehicle and placed on a platform shaker to be shaken overnight. After that, it was centrifuged, filtered, and analyzed in HPLC to determine the amount of drug dissolved in the vehicle. However, if the solubility of the drug in the vehicle is found to exceed the intended donor concentration, this process would not be performed. Various vehicles, including 10% dimethyl isosorbide (DMI), 15% propylene glycol (PG), 15% isopropyl myristate (IPM), 15% dimethyl sulfoxide (DMSO), 20% IPM, 20% PG, 25% PG, 15% DMI, and their combinations in 10 mM phosphate-buffered saline (PBS), were evaluated.

### Preparation of hydrophilic patch

To prepare the base matrix, all components of the patch formulation, excluding the active pharmaceutical ingredients (metoclopramide HCL and sumatriptan succinate), were combined in a scintillation vial with 15 mL of deionized water. This mixture was then placed on a rotary shaker overnight to ensure complete dissolution of all components. The resulting homogeneous solution was carefully poured into 19.63 cm^2^ Petri dishes. The optimal drying time and temperature for the patches were subsequently determined through experimentation, testing a range of temperatures and durations in a drying oven. Once the optimal drying conditions were identified, drug-loaded patches were prepared. This was achieved by adding the pre-determined amounts of metoclopramide HCL and sumatriptan succinate to the previously prepared base solution.

However, incorporating the positively charged metoclopramide HCL and sumatriptan succinate into the initial aqueous solution resulted in an undesirable interaction with the negatively charged polyacrylic acid (PAA) and sodium polyacrylate (SPA) present in the matrix, leading to agglomeration. To mitigate this issue, orthophosphoric acid was added to acidify the solution. This approach was based on the principle that PAA and SPA become protonated and thus lose their negative charge in acidic environments [21]. While this strategy successfully prevented agglomeration, it was observed that a pH of 2.5 was required for complete dissolution, which is too acidic for a topical patch formulation. Consequently, to ensure proper drug incorporation and maintain a suitable pH for skin application, PAA and SPA were removed from the primary patch matrix formulation. To create the final drug-loaded patch solution, all liquid components, including PG, DMI, PEG, glycerin, and water, were combined with the metoclopramide HCL and sumatriptan succinate in a scintillation vial. This mixture was placed on a rotary shaker overnight to ensure thorough mixing. In a separate step, the powdered components, including mannitol, gelatin, and the chosen polymers (such as HPMC, CMC-Na, or PVP) were weighed. The following day, these pre-weighed powdered components were added to the liquid mixture and the entire solution was returned to the rotary shaker for overnight mixing. The resulting homogenous solution was then poured into Petri dishes and dried in an oven under the pre-determined optimal conditions. Once the patches were dried, a 15% (w/w) PAA solution in methanol was uniformly applied to the exposed surface of each patch. This PAA solution was prepared in advance by combining equal weights of 50 wt% and 25 wt% PAA solutions in methanol. For formulations containing PVP, a modified procedure was employed. PVP was initially dissolved in water by heating at 60 °C for 2 h. Following complete dissolution of the PVP, the remaining liquid components (PG, DMI, PEG, glycerin, and water) were added to the PVP solution and the same procedure as described above was applied. Table 1 details the composition of each patch matrix, with percentages showed as w/w, excluding the weight of water.

**Table 1:** Composition of the different hydrophilic patch formulations with polyacrylic acid(PAA) solution in menthol cast in both side

Formulation		PG	DMI	CMC-Na	PVP	HPMC	Glycerin	PEG	Gelatin	Mannitol
**Patch 1**	Percentage	25	10			10	15	30	5	4
	Amount(mg)	510	210			200	306	600	100	80
**Patch 2**	Percentage	25	10	10		2	15	29	5	4
	Amount(mg)	510	210	200		40	300	600	100	80
**Patch 3**	Percentage	25	10		10	20	15	29	5	4
	Amount(mg)	510	210		200	50	300	600	100	80
**Patch 4**	Percentage	25	10		3	9	15	29	5	4
	Amount(mg)	510	210		63	188	300	600	100	80

To identify a suitable backing membrane, a 15% (w/w) PAA solution in methanol was cast onto various membranes, including CoTran™ 9720, CoTran™ 9728, CoTran™ 9702, CoTran™ 9707, CoTran™ 9706, CoTran™ 9722, and ScotchPak™ 9733, using a Gardner casting knife (BYK-AG-4300 series, Columbia, MD, USA). Both 20 µm and 40 µm thicknesses were evaluated for casting the PAA solution. The optimal membrane was selected based on its ability to allow the PAA solution to form a uniform, stable film without any aggregation, and then it was allowed to dry at room temperature for 30 min. Subsequently, the dried patch matrix was removed from the Petri dish using forceps and applied to the chosen backing membrane. To ensure proper adhesion between the patch matrix and the backing membrane, the patch was applied with its bottom side (the non-PAA-coated side) facing the backing membrane which already had a layer of dried PAA on its surface. The assembled patch was then left at room temperature for 2 h to allow for complete adhesion. Next, to identify a suitable release liner, various liners, including ScotchPak™ 9744, ScotchPak™ 1022, 9709 YDS sample roll SKU, and 27,323 PET, were evaluated. Each liner was placed on top of the PAA-coated surface of the assembled patch, and gentle pressure was applied to ensure complete contact without any air gaps. After 30 min, the release liners were removed using forceps. The optimal release liner was chosen based on its ability to detach cleanly from the patch without disrupting the PAA layer or the patch matrix itself.

### Skin source and preparation

Porcine ear skin was obtained and dermatomed using Dermatome 75 µm (Nouvag AG, Goldach, Switzerland). The skin samples were stored at −80 °C until use. Before each study, the skin samples were thawed in 10 mM PBS. The hair on the skin was trimmed with scissors, and the thickness of each sample was measured using a thickness gauge (0–1 in/0–25 mm, Electromatic Equipment Co., Inc., Cedarhurst, NY, USA). Only skin samples with an average thickness of 550 µm were selected for the study.

### Measurement of skin barrier integrity

To ensure the integrity of the skin samples, transepidermal electrical resistance (TEER) measurements were performed using a multi-instrument setup. This setup consisted of silver/silver chloride electrodes, an Agilent 33220 A function generator, and an Agilent 34410 A multimeter (Agilent Technologies, CA, USA). Skin samples were mounted onto vertical Franz diffusion cells, and 300 µL of PBS was added to the donor chamber. The TEER values were measured using protocol previously used in our lab [22]. Skin samples with TEER values below 10 kΩ were excluded from the study. To calculate the TEER, a load resistor (RL = 100 kΩ) was connected in series with the skin sample. A constant voltage drop (Vo = 100 mV) was applied across the circuit. The resulting voltage (Vs) was measured, and the skin resistance was calculated. TEER analysis provides a quantitative assessment of barrier function, with higher values indicating tighter cellular junctions and lower permeability, while lower values reflect compromised barriers and increased permeability [23].

### In vitro permeation testing (IVPT)

In vitro permeation studies were conducted using vertical static Franz diffusion cells (PermeGear, Inc., PA, USA) with a permeation area of 0.64 cm^2^. Dermatomed porcine ear skin was mounted onto the cells, and 10 mM PBS was used as the receptor medium at a volume of 5 mL to maintain sink conditions. The skin surface temperature was maintained at 32 ± 1 °C by regulating the receptor chamber temperature to 37 ± 1 °C, using a water jacket connected to a circulating water bath. At predetermined time intervals (0, 1, 2, 4, 8, 22, and 24 h), 300 µL samples were withdrawn from the receptor chamber and immediately replaced with fresh PBS to maintain consistent receptor volume. The drug concentrations in the collected samples were analyzed using HPLC to evaluate drugs permeation profile. For studies involving chemical enhancers, 100 µL of the drug-loaded solution containing the selected chemical enhancers was applied to the donor chamber. In studies with patches, circular patches with a diameter of 0.62 cm^2^ were punched, and the release liner was removed with forceps. The patches were then applied to the skin surface placed on parafilm, and gentle pressure was applied using a glass rod rolled over the patch to ensure uniform adhesion and contact with the skin. The skin samples with the applied patches were clamped between the donor and receptor compartments of the Franz diffusion cells.

### Characterizations of optimized patches

#### In vitro release testing (IVRT)

In vitro release testing (IVRT) was conducted using vertical static Franz diffusion cells with a permeation area of 0.64 cm^2^. Dialysis membranes were used as the synthetic barrier in this study. After mounting the membrane onto the Franz diffusion cell, a 0.54 cm^2^ section of the patch was placed in the donor compartment. At predetermined time points (0.1, 1, 2, 4, 6, 8, 22, and 24 h), 300 µL of the receptor medium, PBS, was withdrawn and immediately replenished with an equal volume of fresh PBS to maintain sink conditions. The study was conducted in triplicate (*n* = 3). The collected samples were analyzed using HPLC to quantify the amount of drug released over time.

#### Coat weight

To calculate the coating weight of the patch, three samples were punched from different areas of the patch using a 0.62 cm^2^ punch. The punched patches, which included the backing membrane, were weighed. Next, the backing membrane alone was punched three times using the same punch size and weighed separately. The weight of the coating was calculated by subtracting the weight of the backing membrane from the total weight of the patch (including the coating). The results were shown as the mean ± standard error (SE).

#### Drug content and uniformity

Patches were punched three times from different areas using a punch size of 0.62 cm^2^. Each punched sample was placed in a scintillation vial, and 15 mL of 10 mM PBS was added. The vials were then placed on a platform shaker overnight to dissolve. After shaking, the solution was filtered through a 0.22 µm filter, diluted 100 times, and analyzed by HPLC.

#### Tack testing

Tack, a measure of the adhesive's ability to form an initial bond with a different substrate after brief contact and minimal pressure [24] was evaluated using a Texture Analyzer (TA. XT Express, Texture Technologies Corp. and Stable Micro Systems, Hamilton, MA, USA). A stainless-steel cylindrical probe was used to measure the force required for debonding, the positive area, and the separation distance at a speed of 0.5 mm/s, with a return speed of 5 mm/s, and a hold time of 10 s [25].

#### Folding endurance

Folding endurance was evaluated to determine the mechanical strength and flexibility of the transdermal patches. A strip measuring 2 × 2 cm^2^ was repeatedly folded at the same point until it broke. This test was performed in triplicate for each formulation, and the average folding endurance along with the standard deviation was calculated [26].

#### Slide crystallization studies

A slide crystallization study was conducted to determine whether the drug in the patch would crystallize upon vehicle evaporation [27]. The drug-loaded patch solution was cast onto a microscope slide and allowed to dry under a fume hood. The dried sample was then examined under polarized light microscope to assess the presence of any drug crystals.

### Statistical analysis

The results from the studies were analyzed using GraphPad Prism software (GraphPad Software, San Diego, CA; version 9.4.1). Data were presented as mean ± standard deviation (SD) with a sample size of 3 or 4 (n = 3 or 4). One-way ANOVA was used to compare the groups. A significant difference between the test groups was determined when the p-value was less than 0.05 [28].

## Results

### Quantitative analysis

**Fig. 1:**
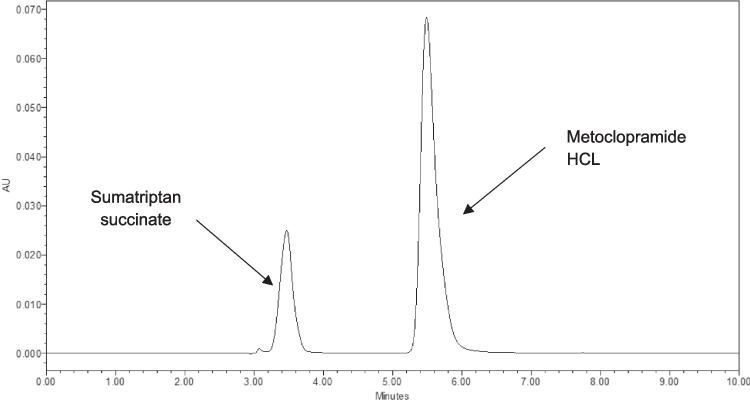
Chromatogram for Metoclopramide HCL and Sumatriptan succinate at a concentration of 50 µg/mL in 10 mM PBS

The method's sensitivity and reproducibility were analyzed through intra-day and inter-day validation. The analysis was conducted three times within a single day and repeated daily over three consecutive days. The limit of detection (LOD) for both compounds was found to be 0.02 µg/mL, while the limit of quantification (LOQ) was determined to be 0.07 µg/mL. As shown in Fig. 1, the peaks for sumatriptan succinate and metoclopramide HCL were observed at 3.21 min and 5.21 min, respectively.

### Saturation solubility

The solubility of metoclopramide HCL and sumatriptan succinate was analyzed in various solutions, including 15% DMI, 25% DMSO, 25% PG, 25% NMP, and 25% IPM, all prepared in 10 mM PBS. It was observed that more than 200 mg of both drugs could dissolve in 1 ml of these solutions. Since this observed solubility significantly exceeded the intended drug concentrations for the donor compartment in subsequent permeation studies, the standard procedure for determining saturation solubility was deemed unnecessary and therefore not conducted. To maintain sink conditions during permeation studies, 10 mM PBS was used as the receptor solution.

### Permeation with different chemical enhancers

**Fig. 2:**
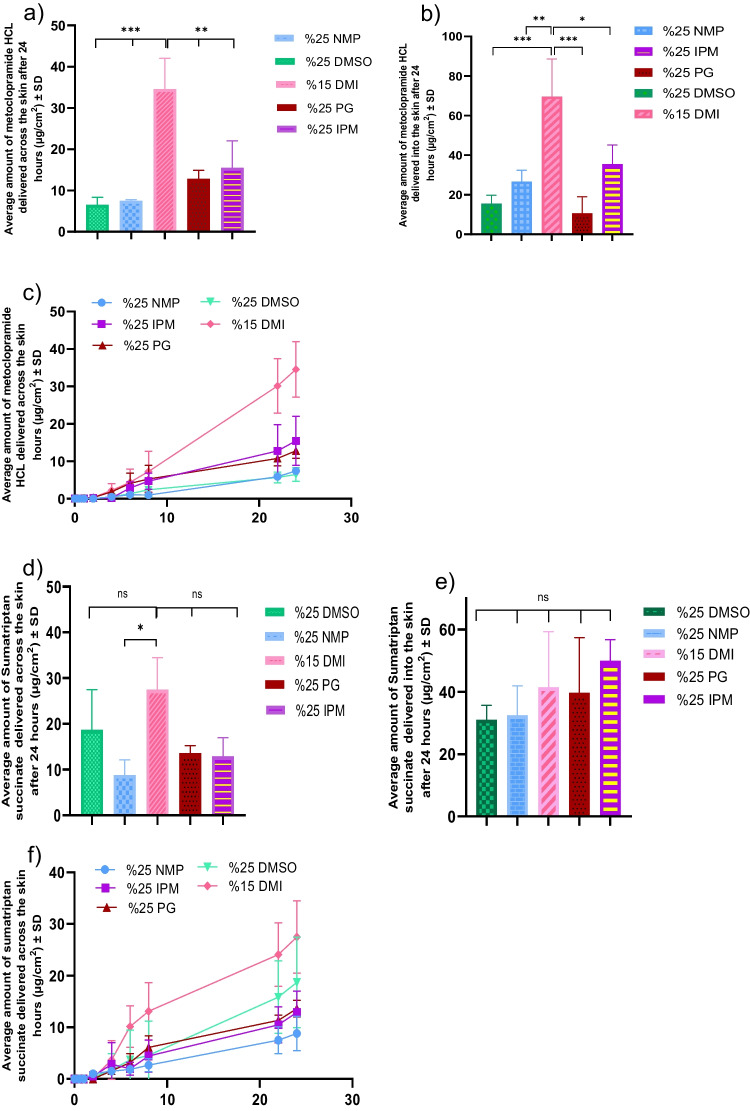
Results from penetration enhancers incorporated IVPT studies (*n* = 4). (**a**) cumulative amount of metoclopramide HCL delivered into the receptor after 24 h; (**b**) amount of metoclopramide HCL delivered into the skin in 24 h. (**c**) Permeation profile of metoclopramide HCL delivered across the skin in 24 h. (**d**) cumulative amount of sumatriptan succinate delivered into the receptor after 24 h; (**e**) amount of sumatriptan succinate delivered into the skin in 24 h. (**f**) Permeation profile of sumatriptan succinate delivered across the skin in 24 h. Statistical analysis performed with one-way ANOVA; ns indicates no significant difference between groups, ** and *** indicate significant difference between groups, *p* < 0.05

For delivery with chemical enhancers, solutions containing 25% w/w NMP, 15% w/w DMI, 25% w/w IPM, 25% w/w PG, and 25% w/w DMSO were prepared in 10 mM PBS. The concentration of sumatriptan succinate in all solutions was 1.13 ± 0.346 mg/mL, and the concentration of metoclopramide HCl was 9.54 ± 0.43 mg/mL. A volume of 100 µL of each solution was applied to a 0.64 cm^2^ donor area. As shown in Fig. 2 and summarized in Table 2, the solution with 15% w/w DMI delivered the highest amount of both drugs, with 27.47 ± 5.73 µg/cm^2^ of sumatriptan succinate and 35.55 ± 6.06 µg/cm^2^ of metoclopramide HCl. For metoclopramide HCl, after 15% w/w DMI, 25% w/w IPM delivered the next highest amount, followed by 25% w/w PG and 25% w/w NMP. The solution with 25% w/w DMSO delivered the lowest amount of metoclopramide HCl. For sumatriptan succinate, the solution with 25% w/w DMSO delivered the next highest amount, followed by 25% w/w PG, 25% w/w IPM, and 25% w/w NMP. Regarding the amount of drug in the skin, there was no significant difference in the amount of sumatriptan succinate retained across the different enhancer groups. However, IPM led to the highest skin deposition of metoclopramide HCl with 69.6 ± 15.45 µg/cm^2^, followed by DMSO and NMP.

**Table 2:** Drug delivery into and across skin with chemical enhancers

Chemical enhancers combinations	Sumatriptan succinate in receptor (µg/cm^2^)	Metoclopramide HCL in receptor (µg/cm^2^)	Sumatriptan succinate in skin (µg/cm^2^)	Metoclopramide HCL in skin (µg/cm^2^)
%25 NMP	8.54 ± 2.83	7.45 ± 0.25	32.48 ± 7.7	26.66 ± 4.6
%25 DMSO	18.7 ± 7.17	48 ± 1.51	31.02 ± 3.8	15.5 ± 3.47
%15 DMI	27.47 ± 5.73	35.55 ± 6.06	41.37 ± 14.66	69.6 ± 15.45
%25 PG	13.61 ± 1.31	12.81 ± 1.68	39.65 ± 14.5	10.62 ± 6.8
%25 IPM	12.88 ± 3.36	15.47 ± 5.35	49.92 ± 5.55	35.44 ± 8.77

### Permeation with various combinations of chemical enhancers

**Fig. 3:**
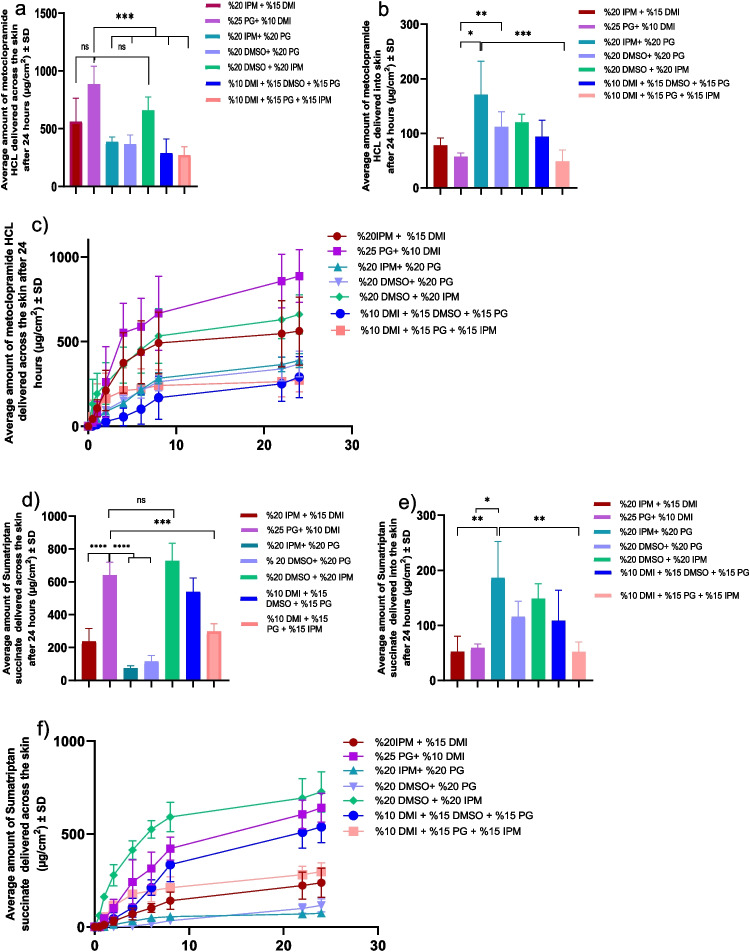
Results from penetration enhancer combination incorporated IVPT studies (*n* = 4). (**a**) cumulative amount of metoclopramide HCL delivered into the receptor after 24 h; (**b**) amount of metoclopramide HCL delivered into the skin in 24 h. (**c**) Permeation profile of metoclopramide HCL delivered across the skin in 24 h. (**d**) cumulative amount of sumatriptan succinate delivered into the receptor after 24 h; (**e**) amount of sumatriptan succinate delivered into the skin in 24 h. (**f**) Permeation profile of sumatriptan succinate delivered across the skin in 24 h. Statistical analysis performed with one-way ANOVA; ns indicates no significant difference between groups, *** and **** indicate significant difference between groups, *p* < 0.05

To analyze the effect of chemical enhancer combinations, several formulations were prepared in 10 mM PBS. These formulations included combinations of 25% NMP, 15% DMI, 25% IPM, 25% PG, and 25% DMSO. The average drug content in all combinations was 78.73 ± 1.31 mg/mL for metoclopramide HCl and 94.38 ± 1.38 mg/mL for sumatriptan succinate. As shown in Fig. 3 and summarized in Table 3, for HCl, the combination of 25% PG + 10% DMI showed the highest delivery, with 886.57 ± 89.99 µg/cm^2^, followed by 20% DMSO + 20% IPM. Lower delivery was observed in the groups with 20% w/w IPM + 20% w/w PG, 20% w/w DMSO + 20% w/w PG, 10% w/w DMI + 15% w/w DMSO + 15% w/w PG, and 10% w/w DMI + 15% w/w PG + 15% w/w IPM. For sumatriptan succinate, the combination of 20% w/w DMSO + 20% w/w IPM exhibited the highest delivery, with 727.87 ± 26.93 µg/cm^2^, followed by 25% w/w PG + 10% w/w DMI. Lower delivery amounts were seen with 20% w/w IPM + 15% w/w DMI. The least delivery was observed in the groups with 20% w/w IPM + 20% w/w PG and 20% w/w DMSO + 20% w/w PG. In terms of skin retention, 20% IPM and 20% PG led to the highest deposition of both drugs in the skin, while the lowest retention was observed with 10% DMI + 15% PG + 15% IPM.

**Table 3:** Drug delivery into and across skin with combinations of chemical enhancers

Chemical enhancers combinations	Sumatriptan succinate in receptor (µg/cm^2^)	Metoclopramide HCL in receptor (µg/cm^2^)	Sumatriptan succinate in skin (µg/cm^2^)	Metoclopramide HCL in skin (µg/cm^2^)
20% IPM + 15% DMI	178.47 ± 45.15	424.44 ± 112.98	52.26 ± 22.8	78.1 ± 10.74
25% PG + 10% DMI	641.29 ± 44.78	886.57 ± 89.99	57.97 ± 5.98	57.5 ± 5.1
20% IPM + 20% PG	55.01 ± 26.38	388.44 ± 23.21	186.38 ± 53.92	171.42 ± 49.6
20% DMSO + 20% PG	116.10 ± 17.75	368.49 ± 38.41	116.03 ± 24.17	119.9 ± 23.9
20% DMSO + 20% IPM	727.87 ± 26.93	660.2 ± 66.97	148.58 ± 22.29	120.4 ± 12.11
10% DMI + 15% DMSO + 15% PG	538.34 ± 48.82	290.93 ± 70.7	108.52 ± 45.03	94.28 ± 24.23
10% DMI + 15% PG + 15% IPM	298.14 ± 27.09	273.57 ± 40.76	52.11 ± 14.7	48.58 ± 17.21

### Preparation of hydrophilic patch

CoTran™ 9728 was selected as the backing membrane, and ScotchPak™ 9744 was chosen as the release liner for all patch formulations. Each patch formulation, incorporating 1000 mg of metoclopramide HCl and 700 mg of sumatriptan succinate, was poured into a petri dish with an area of 19.63 cm^2^. The patches were initially dried in an oven at 65 °C for 6 h, followed by overnight drying at 55 °C, and finally for an additional 2 h at 75 °C. Patch B and C required adjustments to the drying process. Patch C did not dry completely even after overnight drying at 75 °C and was excluded from the study. Patch B successfully dried after extending the drying time to 5 h at 75 °C instead of 2 h. After drying, 700 µL of 15% PAA in methanol was applied to the top of each patch. The patches were then placed in the oven and dried at 60 °C for 2 h. A 15% PAA solution in methanol was cast onto the backing membrane using a casting knife to a thickness of 40 µm.

### In vitro permeation with hydrophilic patches

**Fig. 4:**
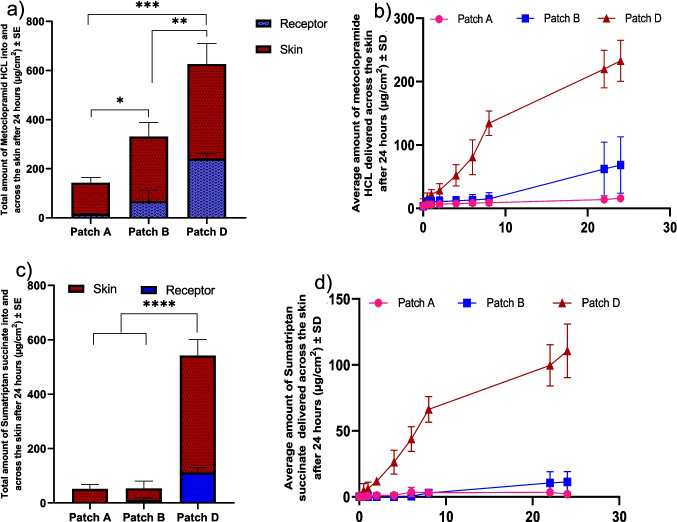
Results from IVPT studies with 3 different patch formulation. (**a**) Distribution of metoclopramide HCL in the skin and receptor compartments. (**b**) Permeation profile of metoclopramide HCL delivered across the skin in 24 h. (**c**) Distribution of sumatriptan succinate in the skin and receptor compartments. (**d**) Permeation profile of sumatriptan succinate delivered across the skin in 24 h. Statistical analysis performed with one-way ANOVA; *** and **** indicate significant difference between groups, *p* < 0.05

Patch D, containing HPMC and PVP, demonstrated the highest permeation, delivering 232.81 ± 18.64 µg/cm^2^ of metoclopramide HCl and 110.64 ± 11.71 µg/cm^2^ of sumatriptan succinate into the receptor and 385.19 ± 68.18 µg/cm^2^ and 429.84 ± 48.24 µg/cm^2^ into the skin, respectively. Patch B, containing CMC-Na and HPMC, delivered 68.33 ± 25.76 µg/cm^2^ of metoclopramide HCl and 11.32 ± 4.58 µg/cm^2^ of sumatriptan succinate to the receptor. Patch A, using only HPMC as the polymer, delivered significantly lower amounts, with 15.99 ± 1.02 µg/cm^2^ of metoclopramide HCl and 2.05 ± 0.52 µg/cm^2^ of sumatriptan succinate. As depicted in Table 4 and shown in Fig. 4, there was a significant difference in the drug delivery of both metoclopramide HCl and sumatriptan succinate from Patch D compared to Patches A and B.

**Table 4:** Drug delivery into skin and receptor with 3 different patch formulations

Formulation	Sumatriptan succinate in receptor (µg/cm^2^)	Metoclopramide HCL in receptor (µg/cm^2^)	Sumatriptan succinate in skin (µg/cm^2^)	Metoclopramide HCL in skin (µg/cm^2^)
Patch A	2.05 ± 0.52	15.99 ± 1.02	49.74 ± 12.61	126.33 ± 17.56
Patch B	11.32 ± 4.58	68.33 ± 25.76	42.16 ± 21.75	262.49 ± 46.83
Patch D	110.64 ± 11.71	232.81 ± 18.64	429.84 ± 48.24	385.19 ± 68.18

### Characterizations of the patches

#### In vitro release testing (IVRT)

**Fig. 5:**
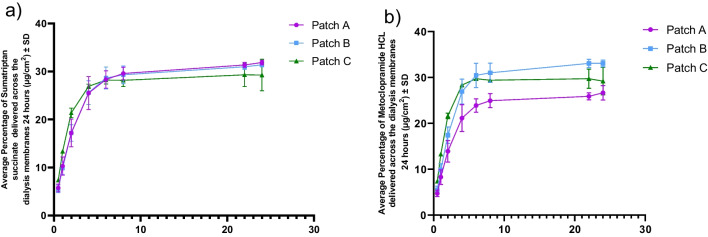
Results from IVRT studies with 3 different patch formulation. (**a**) Cumulative percentage of metoclopramide HCl released into the receptor compartment. (**b**) Cumulative percentage of sumatriptan succinate released into the receptor compartment

Based on IVRT results, the cumulative release of metoclopramide HCl at 24 h was 26.66 ± 0.92% from Patch A, 33.05 ± 0.43% from Patch B, and 27.96 ± 1.90% from Patch D. All three formulations exhibited sustained drug release throughout the 24-h period, with no statistically significant differences among them. For sumatriptan succinate, the percentage of drug released at 24 h was 31.88 ± 0.27% for Patch A, 31.38 ± 0.25% for Patch B, and 27.96 ± 1.90% for Patch D. Similar to metoclopramide HCl, all patches showed extended release of sumatriptan succinate over time with comparable release profiles. As shown in Fig. 5, these results confirm that Patches A, B, and D can deliver both drugs in a sustained manner over 24 h.

#### Coat weight

The coat weight of patches A, B, and D was determined to be 113.73 ± 9.04 mg/cm^2^, 167.18 ± 7.15 mg/cm^2^, and 97.8 ± 3.58 mg/cm^2^, respectively.

#### Drug content and uniformity

The drug content for Patch A was 36.57 ± 0.22% w/w of metoclopramide HCl and 27.79 ± 0.21% w/w of sumatriptan succinate. For Patch B, it was 25.03 ± 0.28% w/w of metoclopramide HCl and 19.26 ± 0.21% w/w of sumatriptan succinate. Patch D had 43.08 ± 0.51% w/w of metoclopramide HCl and 33.26 ± 0.32 w/w of sumatriptan succinate.

#### Tack testing

The absolute force values recorded for Patch A, Patch B, and Patch D were 164.03 ± 6.65 g, 627.01 ± 39.1 g, and 440.03 ± 21.46 g, respectively. Additionally, the positive area and separation distance for each patch were measured, as shown in Table 5.

**Table 5:** Absolute force, positive area, and separation distance measurements for Patch A, Patch B, and Patch D. Values are presented as mean ± standard deviation

Formulation	Measurement of tack		
	Absolute positive force (g)	Positive area (g.s)	Separation distance (mm)
Patch A	164.03 ± 6.65	22.53 ± 3.72	1.96 ± 0.76
Patch B	627.01 ± 39.1	195.30 ± 31.94	10.93 ± 1.22
Patch D	440.03 ± 21.46	137.33 ± 11.58	7.96 ± 2.04

#### Folding endurance

Folding endurance was measured to assess the mechanical strength and flexibility of the transdermal patches. A value greater than 200 indicates the patch’s ability to withstand repeated bending without breaking, reflecting its flexibility and structural integrity for skin application [29]. As shown in Table 2, all batches exhibited folding endurance values within the acceptable range, demonstrating adequate mechanical resistance, durability, and handling stability.

**Table:** 

Formulation	Mean ± Standard Deviation
Patch A	More than 800
Patch B	225 ± 9
Patch D	272 ± 5

#### Slide crystallization studies

**Fig. 6:**
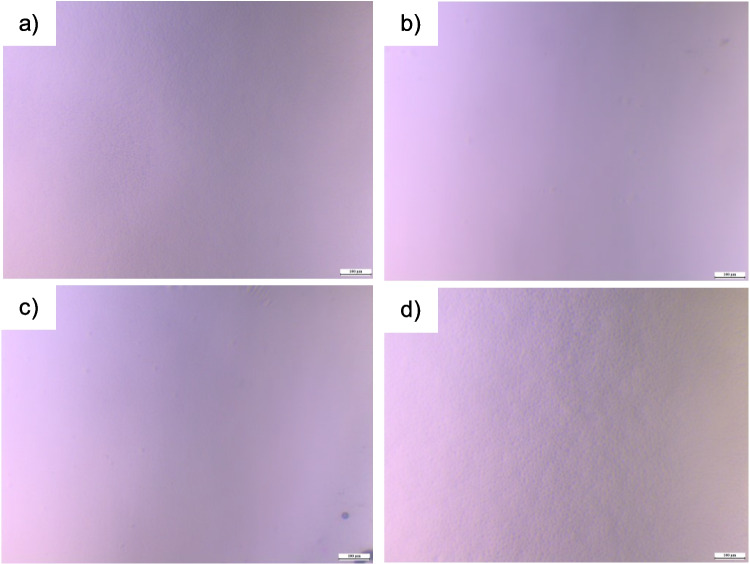
Polarized light microscope images of slide crystallization studies at 20 × magnification. No crystal formation was observed in (**a**) Patch A, (**b**) Patch B, and (**c**) Patch C after drying

To assess the potential for drug crystallization within the transdermal patch, a crystallization study was conducted [30]. As shown in Fig. 6, no crystal formation was observed in any of the patch formulations.

## Discussion

This study aimed to develop a transdermal delivery system for the co-delivery of sumatriptan succinate and metoclopramide HCl to treat migraines. Sumatriptan succinate, a well-established member of the triptan drug class, is widely used for relieving migraine headaches [31]. Metoclopramide HCl has demonstrated pain relief effects comparable to opioids in studies and offers the added benefit of preventing nausea and vomiting, which frequently accompany migraine attacks [32]. However, studies indicate that nausea and vomiting significantly hinder the oral administration of migraine medications, making alternative routes of delivery highly desirable [33]. Among these alternatives, transdermal delivery presents a patient-friendly option as it is non-invasive, eliminates the need for swallowing, and avoids the pain associated with injections. Additionally, by bypassing hepatic first-pass metabolism, transdermal delivery can potentially require a lower drug dosage to achieve the same therapeutic effect as oral medications, thereby reducing the risk of side effects [34]. Therefore, this study explored the potential of delivering both sumatriptan succinate and metoclopramide HCl transdermally.

Transdermal drug delivery typically requires moderate lipophilicity (log P 1–3) and a molecular weight below 500 Da to facilitate passive diffusion through the skin [35]. Metoclopramide HCl, with a log P of 1.79 and a molecular weight of 299 Da, exhibits favorable physicochemical properties for transdermal delivery. Sumatriptan succinate, while meeting the molecular weight criterion at 295 Da, has a log P of 0.74, which is slightly lower than the ideal range [36]. Nevertheless, with appropriate formulation adjustments, such as incorporating chemical penetration enhancers, both drugs are suitable candidates for transdermal delivery.

Sumatriptan succinate is currently available in both oral and injectable formulations. The oral dosage ranges from 25 to 100 mg, while subcutaneous injection doses typically range from 4 to 6 mg [37]. Metoclopramide HCl is currently available in the market as an oral dosage form, with recommended doses of either 5 mg or 20 mg [38]. In this study, the target dose for sumatriptan succinate was calculated as 66 µg/cm^2^ for a 60 cm^2^ patch, equivalent to a 4 mg subcutaneous injection. Similarly, the target dose for metoclopramide HCl was determined to be 133.33 µg/cm^2^ for a 60 cm^2^ patch, corresponding to a 10 mg oral dose.

Functional excipients, such as penetration enhancers and solubilizers, are known to influence skin barrier properties. These excipients may alter drug solubility, or disrupt the lipid packing within the stratum corneum, which could create pathways for drug molecules to pass through the skin [39, 40]. Previous research, including our own studies, suggests that incorporating chemical enhancers into transdermal delivery systems can support drug permeation across the skin barrier [41]. In preliminary solubility studies, both metoclopramide HCl and sumatriptan succinate showed good solubility in PBS and PBS solutions with various chemical enhancers. All the chemical enhancers were used within the concentration range specified in the FDA's Inactive Ingredient Guide (IIG). In vitro permeation studies using dermatomed porcine ear skin showed that both drugs could permeate the skin. Notably**,** a formulation that had DMI incorporated into PBS delivered higher amounts of sumatriptan succinate and metoclopramide HCl compared to other chemical enhancers. DMI's primary function is to enhance the delivery of active ingredients deeper into the skin by slightly disrupting the skin barrier, thereby improving drug absorption and efficacy [42]. However, despite its effectiveness in increasing drug permeation, the amount of drug reaching the receptor compartment was insufficient to meet the therapeutic target. The concentration gradient is a key factor influencing the effectiveness of topical and transdermal drug delivery systems. An increased degree of drug saturation in the formulation enhances the concentration gradient across the skin barrier. This elevated gradient drives the movement of drug molecules from the formulation into and through the skin layers, improving overall drug permeation and delivery efficiency [43, 44].

To enhance drug delivery in the subsequent study, a combination of chemical enhancers was employed, and the drug concentration was increased. Different classes of chemical enhancers were selected for their unique properties and mechanisms of action. These mechanisms involve interactions with lipids and proteins in the stratum corneum, as well as modifications to the drugs solubility [45, 46]. Combining multiple enhancers in this way creates a synergistic effect, which can significantly improve drug permeation. Moreover, using enhancers at lower individual concentrations reduces the risk of skin irritation or adverse effects that might arise with higher concentrations of a single enhancer [47]. Among the tested combinations, 25% PG + 10% DMI delivered the highest amount of metoclopramide HCl, while 20% DMSO + 20% IPM delivered the highest amount of sumatriptan succinate, reaching 627.87 ± 26.93 µg/cm^2^. Given that the therapeutic target for metoclopramide HCl is higher than that for sumatriptan succinate, the combination of 25% PG + 10% DMI was selected to incorporate in patch formulation.

Transdermal patches offer a non-invasive and convenient method for drug administration [48]. Polymers play an important role in these patches, providing essential mechanical properties and enabling the controlled release of drugs [49]. Therefore, selecting the appropriate polymer matrix is important for developing effective transdermal drug delivery systems. Polymers not only regulate drug release but also enhance skin permeation and can support targeted delivery [50]. HPMC, a derivative of cellulose ether, is commonly used in controlled drug delivery systems. Its non-toxic, biocompatible, and biodegradable nature, along with its thickening, gelling, and swelling properties, makes it an excellent choice for creating controlled-release formulations. CMC-Na, an anionic polymer, is known for its swelling properties and uniform viscosity. It forms a protective film on the skin, helps retain moisture, and is commonly used in topical and transdermal formulations to achieve sustained drug delivery [51]. PVP, a hydrophilic polymer, has a wide range of applications in pharmaceuticals, cosmetics, and food products. It is highly soluble in water and organic solvents, biocompatible, and improves drug solubility. Additionally, PVP prevents drug crystallization and enhances patch adhesion, making it suitable for transdermal drug delivery systems [52]. In this study, four hydrophilic patch formulations were developed using different polymer combinations to determine the most effective matrix for delivering metoclopramide HCl and sumatriptan succinate through the skin into the receptor. To improve the adhesion of the patch to the skin, PAA and SPA were included in the patch formulation. Both PAA and SPA are synthetic high-molecular-weight polymers of acrylic acid. Both are anionic polymer and have side chains that ionize at neutral pH, resulting in a negative charge [53]. However, metoclopramide HCl and sumatriptan succinate, being the salt forms of their respective drugs, carry a positive charge in solution [54, 55]. The interaction between the negatively charged polymers and the positively charged drugs led to the formation of clumps during the preparation of the patch. To address this issue, PAA and SPA were removed from the initial patch formulation and added later by dissolving them in methanol and applying them to the dried patch. This is because PAA and SPA exhibit reduced negative charge in methanol compared to water [56], which minimized interactions with the positively charged drugs. Additionally, ionic chemicals possess less charge in their dry forms [57, 58], further reducing the likelihood of undesirable interactions.

Based on previous studies, varying polymer ratios can significantly influence drug delivery outcomes. For example, a ratio of 5:1 of CMC-Na to HPMC was reported to deliver the highest amount of drug in one study [59]. In another, a ratio of 8:2 of PVP to HPMC showed the best results [60], whereas a 1:3 ratio of PVP to HPMC was most effective in different research[61].

In this study, a patch formulation with an 8:2 ratio of HPMC to PVP (Patch C) was found to be too sticky to detach from the Petri dish, resulting in its exclusion from further testing. Among the remaining formulations, Patch B exhibited the highest adhesiveness based on the tack testing results. It demonstrated the highest absolute positive force, positive area, and separation distance, indicating more adhesive strength and force required for its’ detachment [27]. However, the inclusion of CMC-Na in the formulation resulted in lower drug delivery. This reduced performance may be due to the negative charge of CMC-Na, which likely interacted with the positively charged drugs, hindering their release and limiting their delivery efficiency. Patch A, made with only HPMC, delivered the least amount of drug. This may be because HPMC forms a dense matrix and has high viscosity, both of which can slow down drug release [62]. In contrast, Patch D, formulated with a 1:3 ratio of PVP to HPMC, demonstrated the highest drug delivery for both metoclopramide HCl and sumatriptan succinate. In fact, patch D successfully achieved the target drug delivery for both drugs simultaneously within 8 h, delivering 134.28 ± 11.12 µg/cm^2^ of metoclopramide HCl and 66.30 ± 5.61 µg/cm^2^ of sumatriptan succinate.

Based on IVRT results, all patches released approximately 29% of metoclopramide HCl and 30% of sumatriptan succinate over 24 h, with no significant difference observed among the formulations. This can be attributed to the highly hydrated conditions of the test system, where the pre-soaked dialysis membrane ensured uniform moisture availability and minimizing the impact of formulation-dependent properties such as viscosity, ionic interactions, or matrix architecture [63]. Under conditions of abundant moisture, drug diffusion is driven primarily by concentration gradients rather than formulation barriers [35]. In contrast, IVPT more accurately simulates physiological skin conditions, where limited moisture and a structured lipid–protein barrier amplify differences in formulation behavior. These findings are consistent with prior reports emphasizing that IVRT is best suited for quality control and batch comparison, whereas IVPT provides greater predictive value for transdermal drug delivery performance [64].

While the results of this study are encouraging, additional research is needed to improve the clinical relevance and applicability of the findings. The in vitro studies were conducted using dermatomed porcine ear skin, which may not fully represent human skin physiology, highlighting the need for validation in human skin models. Long-term stability and large-scale manufacturing aspects were also not explored. Scaling up transdermal patch production poses challenges related to drug permeability, formulation stability, and manufacturing consistency. The current study offers a preliminary basis for exploring transdermal patches as a potential method for the co-delivery of metoclopramide HCl and sumatriptan succinate, aiming to provide a patient-friendly option for migraine management.

## Conclusion

In conclusion, this study explored the development of transdermal patches for the co-delivery of metoclopramide HCl and sumatriptan succinate as a potential alternative to conventional oral and injectable formulations for migraine management. The findings demonstrated that a combination of chemical enhancers and optimized polymer matrix significantly improved drug delivery. Among the tested formulations, Patch D, composed of a 1:3 ratio of PVP to HPMC, achieved the target delivery of both drugs within 8 h, highlighting its potential for therapeutic application.

## Data Availability

The datasets generated during and/or analyzed during the current study are included in the manuscript.
